# A New Sensor for Methyl Paraben Using an Electrode Made of a Cellulose Nanocrystal–Reduced Graphene Oxide Nanocomposite

**DOI:** 10.3390/s19122726

**Published:** 2019-06-18

**Authors:** Wan Elina Faradilla Wan Khalid, Mohamad Nasir Mat Arip, Latifah Jasmani, Yook Heng Lee

**Affiliations:** 1 School of Chemical Sciences and Food Technology, Faculty of Science and Technology, Universiti Kebangsaan Malaysia, Bangi 43600, Selangor, Malaysia; wan_elina@uitm.edu.my; 2 Faculty of Applied Sciences, Universiti Teknologi MARA Negeri Sembilan, Kuala Pilah Campus, Pekan Parit Tinggi, Kuala Pilah 72000, Negeri Sembilan, Malaysia; 3 Forest Products Division, Forest Research Institute Malaysia, Selangor 52109, Malaysia; mnasir@frim.gov.my (M.N.M.A.); latifah@frim.gov.my (L.J.)

**Keywords:** methyl paraben, cellulose nanocrystal, reduced graphene oxide, electrochemical sensor

## Abstract

A new cellulose nanocrystal–reduced graphene oxide (CNC–rGO) nanocomposite was successfully used for mediatorless electrochemical sensing of methyl paraben (MP). Fourier-transform infrared spectroscopy (FTIR) and field-emission scanning electron microscopy (FESEM) studies confirmed the formation of the CNC–rGO nanocomposite. Cyclic voltammetry (CV) studies of the nanocomposite showed quasi-reversible redox behavior. Differential pulse voltammetry (DPV) was employed for the sensor optimization. Under optimized conditions, the sensor demonstrated a linear calibration curve in the range of 2 × 10^−4^–9 × 10^−4^ M with a limit of detection (LOD) of 1 × 10^−4^ M. The MP sensor showed good reproducibility with a relative standard deviation (RSD) of about 8.20%. The sensor also exhibited good stability and repeatability toward MP determinations. Analysis of MP in cream samples showed recovery percentages between 83% and 106%. Advantages of this sensor are the possibility for the determination of higher concentrations of MP when compared with most other reported sensors for MP. The CNC–rGO nanocomposite-based sensor also depicted good reproducibility and reusability compared to the rGO-based sensor. Furthermore, the CNC–rGO nanocomposite sensor showed good selectivity toward MP with little interference from easily oxidizable species such as ascorbic acid.

## 1. Introduction

Parabens or esters of *p*-hydroxybenzoic acid are widely used as antimicrobial preservatives in cosmetics, pharmaceuticals, and personal care products due to their low toxicity [1]. Their properties of low cost, reasonable solubility in water, a long history of safe applications, and no significant taste or odor represent the reasons for their widespread applications [2]. Nevertheless, the safety of paraben as a preservative, especially in cosmetic products such as underarm deodorants and antiperspirant, was questioned when paraben was found in human breast tissue [3]. Cosmetic Directive 76/768/EEC annex VI limited the maximum level of parabens in cosmetics to 0.4% for one ester and 0.8% for a mixture of esters [4]. Conventional analytical methods such as gas chromatography (GC) [5], high-performance liquid chromatography (HPLC) [6], and capillary electrophoresis [7] are extensively used for paraben detection. However, the development of electrochemical sensors for paraben detection is attractive due to the possibility of simple, rapid, and in situ analysis compared with conventional analytical methods.

To date, several nanocomposite-based sensors for detection of paraben were actively explored. There are many electrochemical sensors for paraben detection including molecular imprinted polymers (MIP) for propyl paraben (PP) [8] and total paraben amount [9], fullerene nanorods for an ethyl paraben (EP) sensor [10], and indium oxide (In_2_O_3_) nanobricks for butylparaben (BP) [11]. The first biosensor for paraben was fabricated by Hajian et al., where methyl paraben (MP) was determined using hemoglobin with a limit of detection (LOD) of 25 nM [12]. Very few studies of sensors for paraben involved the use of graphene oxide (GO). So far, there are only two studies that used GO for paraben determination. Graphene (GR) nanocomposite was used together with polyvinylpyrrolidone (PVP) or polyaniline (PANI) for the determinations of five paraben compounds, where GR was used to increase the surface area and offered a higher sensitivity to the sensor, whilst PVP and PANI were added as dispersing agents and to increase the conductivity of the electrodes. Paraben compounds were determined with amperometry after separation with the HPLC technique [13]. In another study, Piovesan et al. used a reduced GO (rGO)–gold nanoparticle (AuNP) nanocomposite for MP detection. Chitosan was used for the GO reduction process and for rGO and AuNP stabilization. The rGO–AuNP nanocomposite provided a more sensitive method for MP detection due to the synergistic effect between both rGO and AuNP [14].

Herein, we developed an electrochemical sensor for the determination of MP by using a cellulose nanocrystal (CNC)–rGO nanocomposite. MP was used as a model to demonstrate the sensor performance as the sensor is unable to distinguish different types of paraben compounds. MP and PP are frequently used in cosmetics due to the synergistic effects when both compounds are mixed. However, the estrogenic activity increases with an increase in the length of the paraben alkyl group, which makes MP the least estrogenic compound [15]. Until now, there are only two studies that used a similar composite for sensor applications. The applications of CNC–rGO nanocomposites were mostly related to measurements of resistance and capacitance change, e.g., for proximity [16] and humidity sensing [17]; however, the nanocomposite is yet to be used for MP detection. The CNC–rGO nanocomposites that were used in this study involved simple preparation steps of sonicating and drop coating. In a study by Mauro et al. [18], they also used almost a similar approach for a ZnO/polymethyl methacrylate (PMMA) nanocomposite by simply sonicating and casting it in a petri dish.

In addition to its good film-forming ability, good biocompatibility, and non-toxic properties, CNCs were used to reduce or avoid adsorption of MPs onto the rGO surface. Reduced GO is known for its capability to adsorb organic compounds through π–π stacking on its surface. Without the random presence of CNC on the rGO surface, MPs may easily adsorb onto the rGO surface even though rGO also has a good film-forming property [19]. The differences in analytical performance between rGO and the rGO–CNC nanocomposite modified electrodes toward MP detection were also examined. The CNC–rGO nanocomposite presented good reusability and higher selectivity when compared to rGO modified electrodes.

## 2. Materials and Methods

### 2.1. Materials and Chemicals

CNC that was synthesized from Kenaf and rGO that was previously reduced from GO using hydrazine were provided by the Forest Products Division, Forest Research Institute Malaysia (FRIM) and Universiti Malaysia Pahang (UMP), respectively. Potassium hexacyanoferrate (III) (ACS Reagent, ≥99%) and salicylic acid (SA) were purchased from Sigma-Aldrich. Ethyl alcohol (95%), ascorbic acid (AA), and citric acid were obtained from SYSTERM. Methyl 4-hydroxybenzoate was acquired from Fluka. Benzoic acid (BA) was purchased from BDH. Potassium dihydrogen phosphate and potassium phosphate dibasic anhydrous from Sigma-Aldrich were used to prepare a 0.05 M phosphate buffer solution (PBS), pH 5–8. The deionized water used for solution preparation was from a Thermo Scientific™ Barnstead™ Water System (18.2 MΩ·cm).

### 2.2. Instrumentation

The characterization of the individual material and nanocomposite was recorded using a field-emission scanning electron microscope (FESEM; Merlin, Zeiss, Germany). Fourier-transform infrared (FTIR) spectra were recorded using a Perkin Elmer Spectrum 400 FT-IR. Electrochemical characterization was carried out using cyclic voltammetry (CV) and differential pulse voltammetry (DPV), while electrochemical impedance spectroscopy (EIS) was performed using Autolab PGSTAT 12 Potentiostat (NOVA 1.10 software, Utrecht, The Netherlands). A conventional three-electrode system was used in which the screen-printed electrode (SPE) (Scrint Technology (M) Sdn. Bhd; Kedah, Malaysia) modified with CNC–rGO was employed as the working electrode, with the glassy carbon electrode as the counter electrode and Ag/AgCl as the reference electrode. 

### 2.3. Preparation of the Nanocomposite and Modified Electrode

The CNC–rGO nanocomposite was prepared by dispersing and ultrasonicating 1 mg of rGO in 200 µL of ethanol and 1 mg of CNC in 500 µL of deionized water. The rGO and CNC dispersions were then mixed and sonicated for 1 h. The supernatant was removed by using centrifugation to get the final CNC–rGO nanocomposite. The nanocomposite CNC–rGO was re-dispersed in 200 µL of ethanol. For preparation of the modified electrode, about 5 µL of CNC–rGO nanocomposite was drop-coated onto the electrode surface, and it was left to dry at room temperature before use.

### 2.4. Electroanalytical Characterization

The characterization of the bare and modified electrode was performed using CV in 10 mM K_3_Fe(CN)_6_ in a 0.1 M KCl solution at a scan rate of 0.01 V/s. The effect of scan rate for rGO and CNC–rGO modified electrodes was measured from 10–80 mV/s. EIS was also carried out in a 10 mM [Fe(CN)_6_]^3−^/[Fe(CN)_6_]^4−^ solution in 0.1 M KCl with a frequency range of 10,000 Hz, an amplitude of 0.01 V, and a potential of 0.4 V.

### 2.5. Electrochemical Sensing of the MP

All electrochemical measurements were carried out in PBS pH 7.0 (0.05 M) containing MP except for the pH study. All optimizations of the electroanalytical responses were measured using DPV at a fixed time of 120 s. The measurements were recorded in the potential range of +0.4 to +1.2 V with a scan rate of 0.01 V/s.

The effect of different ratios of CNC:rGO nanocomposite toward 5 × 10^−3^ M MP detection was investigated using six different ratios (0.5:1, 1:1, 2:1, 3:1, 1:2, and 1:3). The modified CNC–rGO electrodes were prepared by drop-coating 5 µL of the nanocomposite from each ratio, and the highest current response was used for the subsequent optimization.

The effect of pH was performed by preparing a 5 × 10^−3^ M MP solution in PBS (0.05 M) with pH 5, pH 6, pH 7, and pH 8. The determination of the linear response range was carried out by plotting the calibration curve of the sensor current response toward MP in the concentration range of 0–9 × 10^−3^ M.

The reproducibility study was carried out by measuring the results of five different rGO and CNC–rGO nanocomposite modified electrodes toward the presence of 4 × 10^−4^ M MP. The relative standard deviation (RSD) value was calculated after current measurement. 

The repeatability study of both rGO and CNC–rGO nanocomposite modified electrodes was completed using one single electrode, and the current produced was measured continuously and alternately in 3 × 10^−4^ M and 7 × 10^−1^ M MP. The RSD value was reported after each measurement.

The stability study was performed by measuring the current toward 5 × 10^−3^ M MP on every day and then for the duration of one week. For each measurement, three electrodes were used. The stability study was carried out and continued for 28 days.

The effect of potential interference on the rGO and CNC–rGO nanocomposite modified electrodes was tested in 4 × 10^−1^ M ascorbic acid, benzoic acid, salicylic acid, and citric acid. The effect of possible interference species was also investigated using a mixed solution method at different concentration ratios.

### 2.6. Application of the Sensor to the Real Sample Analysis

Analysis of the real sample was carried out using a standard addition method. The procedure for real sample analysis was the same as previously reported by Qurashi et al. with some modifications [11]. About 0.1 g of *Aloe vera* cream was weighed and dissolved in 25 mL of ethanol. Sonication was then performed for 30 min, followed by centrifugation for 20 min at 10,000 rpm. The supernatant was then dissolved in 100 mL of 0.05 M PBS pH 7.0 before measurement.

## 3. Results and Discussion

### 3.1. Characterization of the CNC, rGO, and CNC–rGO Nanocomposite

**Figure 1:**
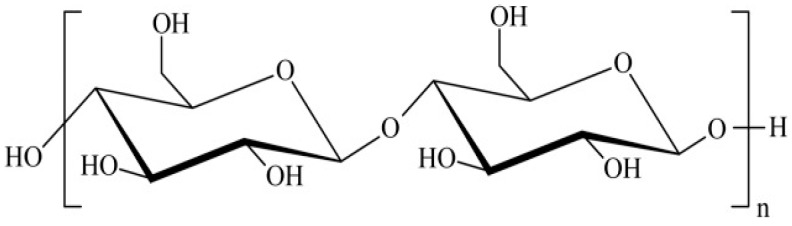
The structure of cellulose (Mariano et al. [32]).

Figure 1 shows the chemical structure of CNC, while Figure S1 (Supplementary Materials) shows the FTIR spectra for CNC, rGO, and CNC–rGO nanocomposite. The FTIR spectrum can be used to confirm the formation of the CNC–rGO nanocomposite by identifying the functional group. CNC displays an OH stretching band at 3329 cm^−1^ and a peak at 2895 cm^−1^ due to C–H stretching [20]. At 897 cm^−1^, it shows the glycosidic bond between glucose units [21], while the band at 1030–1160 cm^−1^ displays the glucose ring [22]. GO is an insulator that has many oxygen functional groups at the basal and edge plane, which should be converted for conductive rGO [23,24]. There is no peak at around 1700 cm^−1^ due to the removal of the carbonyl group (C=O). The FTIR spectrum demonstrates no oxygen-containing group for rGO [25]. The absence of the peak at 1130 cm^−1^ indicates no C–O bond (epoxy or alkoxy) [26], while peaks at 1622 cm^−1^ refer to the C=C aromatic bond [23]. The peak at 870 cm^−1^ indicates C–C vibration [25]. The FTIR spectrum of the nanocomposite CNC–rGO is almost the same as the FTIR spectrum for CNC. This may be due to the CNC at the rGO surface, which resulted in the functional group of CNC being detected although the nanocomposite formed was black in color, as depicted in Figure S1 (Supplementary Materials).

**Figure 2:**
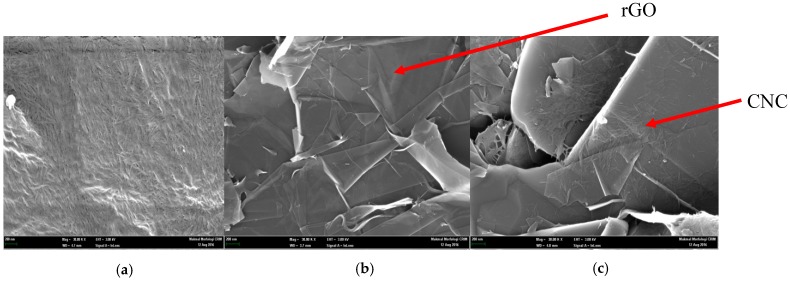
Field-emission scanning electron microscopy (FESEM) image of the (**a**) cellulose nanocrystal (CNC), (**b**) reduced graphene oxide (rGO), and (**c**) nanocomposite CNC–rGO at 30,000× magnification.

The morphologies of the CNC, rGO, and nanocomposite CNC–rGO were characterized using FESEM. The FESEM image (Figure 2a) shows the individual rod-like structure of CNC and some aggregations [27]. Figure 2b confirms the wrinkled and folded characteristic of rGO [28,29,30], while, for nanocomposite CNC–rGO, it can be clearly seen that CNC is likely to randomly cover the surface of rGO (Figure 2c). This happens due to the uncovered flat surface of rGO, which can adsorb suitable nanomaterials [31].

### 3.2. Electroanalytical Characterization of the Modified Electrode

The ability of the modified electrode surface to transfer electrons was characterized using the redox mediator K_3_[Fe(CN)_6_]. CV of bare SPE and modified electrodes was recorded and is depicted in Figure S2 (Supplementary Materials), displaying a quasi-reversible redox voltammogram. A large peak-to-peak separation (ΔEp = 203 mV), as presented in Table S1 (Supplementary Materials), shows poor conductivity of the bare electrode. The current signal for the CNC modified electrode toward the redox pair Fe^2+^/Fe^3+^ was lower than that of the bare electrode. This may be attributed to the insulator properties of CNC, whereby it blocked the electron transfer at the electrode surface resulting from the difficult accessibility of the [Fe(CN)_6_]^3−^ [33,34]. A higher current was demonstrated by rGO and rGO–CNC modified electrodes due to the presence of rGO, which possesses high electrical conductivity [35]. Lower values of peak-to-peak separation between oxidation and reduction peaks of modified electrodes as compared to SPE/CNC show a fast electron transfer of the modified electrodes [36]. However, rGO–CNC (anodic current (Ipa) = 97.63 µA) showed a lower current value as compared to rGO (Ipa = 121.77 µA). 

A scan rate study was performed for rGO and CNC–rGO modified electrodes in the range of 10–80 mV/s as displayed in Figure S3 (Supplementary Materials). A proportional relationship in the plot of peak current oxidation and reduction (Ipa/Ipc) versus the square root of the scan rate indicated that the reaction was a diffusion-controlled process that occurred at the electrode surface [37]. The electrochemical active surface area of the electrodes (rGO and CNC–rGO) was determined based on the Randles–Sevcik equation (Equation (1)) at a scan rate of 10 mV/s [38]. Thus, the electrochemically active surface areas were found to be 0.1887 cm^2^ and 0.1456 cm^2^ for rGO and CNC–rGO, respectively. Based on this value, rGO was found to have a much higher electrochemically active area compared to CNC–rGO.

I_p_ = (2.69 × 10^5^)*n*^3/2^*AcD*^1/2^*v*^1/2^, (1)

where I_p_ = peak current (A), *n* = number of electrons in the reaction (1 for K_3_[Fe(CN)_6_]), *A* = electrochemically effective surface area of the working electrode (cm^2^), *c* = concentration of the reactant (mol∙cm^−3^), *D* = diffusion coefficient (7.6 × 10^−6^ cm^2^∙s^−1^ for K_3_[Fe(CN)_6_), and *v* = scan rate (V∙s^−1^).

EIS was used to characterize and measure the charge transfer process that occurred at the solution–electrode or solution–modified electrode interface. The EIS spectrum in the form of a Nyquist plot consists of a linear part at a lower frequency, referring to the diffusion-controlled process, and a semicircular part at a higher frequency, which shows the electron transfer-limited process [39]. The diameter of the semicircle is equal to the electron transfer resistance (R_ct_), where it refers to the difficulty of the electron transfer between solution and electrode by the redox couple ferrocyanide/ferricyanide [40,41]. Bare SPE showed a small semicircle in the Nyquist plot, where the R_ct_ value was 7052 Ω, as presented in Figure S4 (Supplementary Materials) [42]. The CNC modified electrode showed that the diameter of the plot increased as compared to bare SPE, together with an increase in R_ct_ value to 15,537 Ω. This was due to the CNC that blocked the transfer of electrons. With the presence of rGO, the diameter was reduced significantly due to its conductive properties which promote electron transfer. When the electron transfer is rapid, the Nyquist plot is dominated by a straight line, while, for slow electron transfer, the semicircular part predominates [43]. For the rGO and CNC–rGO modified SPEs, there was no significant difference in the Nyquist plots, which clearly showed that rGO improved the electron transfer characteristics at the electrode surface.

### 3.3. Electroanalytical Behavior of the MP Sensor

**Figure 3:**
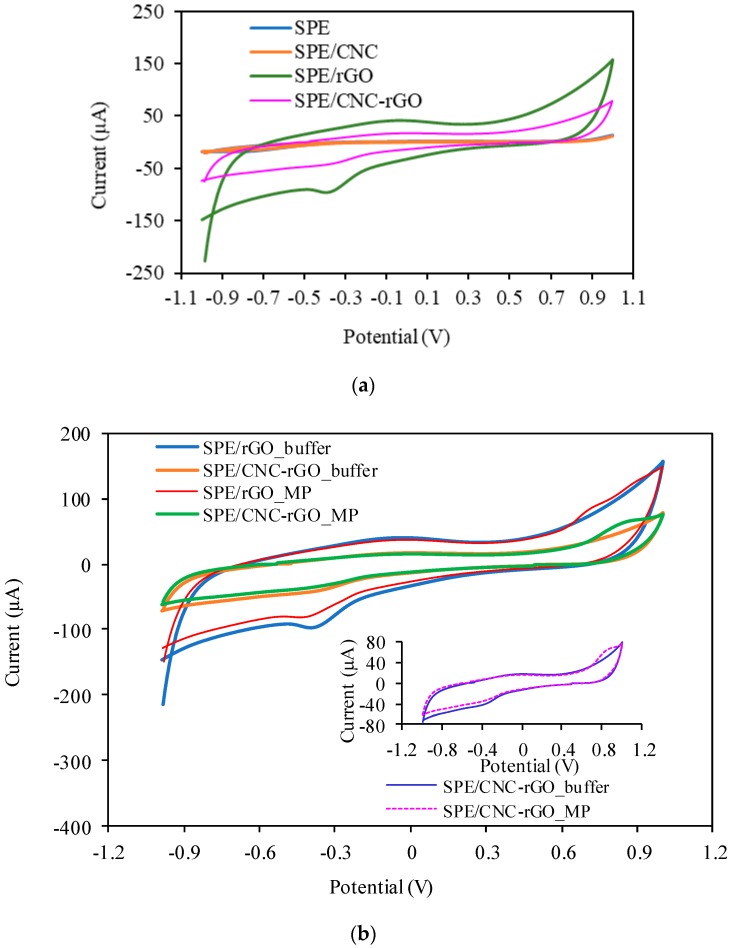
(**a**) Response of the modified electrodes in PBS pH 7.0 (0.05 M); (**b**) response of the modified electrodes in PBS pH 7.0 (0.05 M) and 7 × 10^−4^ M MP.

**Figure 4:**
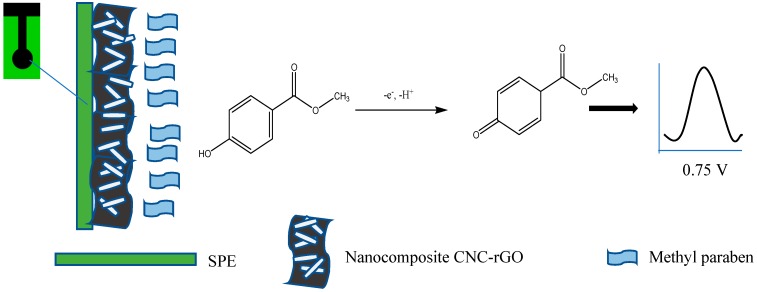
Schematic diagram of the sensor fabrication and MP detection.

The electrochemical behavior of the modified electrodes in the absence of MP (only in 0.05 M PBS pH 7.0) was examined. There was a reduction peak at about −0.3 V; thus, this was not related to the electrochemistry of MP and may be attributed to that of rGO (Figure 3a). In the presence of 7 × 10^−4^ M MP, the modified electrode displayed a significant current increase. However, the SPE/CNC–rGO nanocomposite demonstrated a lower current value than SPE/rGO. A single oxidation peak at a potential of ~0.8 V appears in Figure 3b, which is the result of catalytic oxidation of the phenolic group in the MP to form benzoquinone, while no reduction peak indicates that the reaction was irreversible [11]. Figure 4 displays the diagram for electrochemical sensor fabrication and MP detection.

I_p_ = 697.85*v*^1/2^ − 3.0583 (*R*^2^ = 0.9909) (2)

I_p_ = 390.11*v*^1/2^ + 3.1676 (*R*^2^ = 0.9934) (3)

Figure S5 (Supplementary Materials) illustrates the plot of Ipa versus square root of the scan rate. A linear relationship was demonstrated for the redox reaction of MP for both the rGO modified electrode (Equation (2)) and the nanocomposite CNC–rGO modified electrode (Equation (3)), indicating the diffusion-controlled process. The oxidation peak shifted to a more positive potential when the scan rate was increased, and this confirmed that the oxidation of MP was an irreversible process [44]. Furthermore, the plot of log Ipa versus log scan rate in Figure S6 (Supplementary Materials) also demonstrates a linear relationship. The oxidation that happened on the surface of the electrode was due to an adsorption process if the slope value of the linear relationship between log Ipa and log scan rate was in the range of 0.5–1.0 [45]. Based on the plot of log Ipa versus log scan rate, the slope values for rGO and CNC–rGO nanocomposite modified electrodes were 0.52 and 0.48, respectively. Thus, the oxidation process that happened on the rGO modified electrodes was due to the adsorption process, while that on the CNC–rGO modified electrode may have been due to the diffusion process. The contradictory behavior of the electroanalytical processes which happened on the surface of the electrode, especially for the rGO modified electrode, may be due to the possibility of a combination of diffusion and adsorption processes which depended on the scan rate [46].

Based on the effect of the cycle in Figure S7 (Supplementary Materials), it is indicated that both rGO and CNC–rGO nanocomposite showed a reduced current with the increase of cycles. The electrode modified with rGO gave a similar current value to background at cycle 10, while the CNC–rGO modified electrode gave a similar value at cycle 8. However, rGO showed a higher current reduction as compared to the CNC–rGO nanocomposite. After eight cycles, rGO exhibited a 17-µA current reduction, while CNC–rGO displayed a 12-µA current reduction. This may have been due to the adsorption of MP onto the rGO surface, while the presence of CNC on the rGO surface may have reduced the MP adsorption onto its surface. The reduced peak currents when the cycle was increased for CNC–rGO nanocomposite modified electrodes may be attributed to the adsorption of the MP onto the rGO surface which was not covered by CNC.

For an irreversible electrode process, the Laviron theory defines Ep as follows:

Ep = E^0′^ + (2.303RT/*αn*F)log (RTk_0_/*αn*F) + (2.303RT/*αn*F)log *v* (4)

where α = transfer coefficient, k_0_ = standard heterogeneous rate constant of the reaction, n = number of electrons transferred, *v* = scan rate, E^0′^ = formal redox potential, R = gas constant (8.314 J·mol^−1^·K^−1^), T = room temperature (298.15 K), and F = Faraday constant (96,485.34 C∙mol^−1^).

The value of *αn* can be calculated using the slope from Epa versus log *v* using the following equation: 2.303RT/*αn*F. Figure S8a (Supplementary Materials) displays the plot of Epa versus log *v* for both rGO and nanocomposite CNC–rGO. The equation can be expressed as Epa (V) = 0.0901log *v* (V/s) + 0.9974 for rGO, and Epa (V) = 0.1331log *v* (V/s) + 1.0628 for CNC–rGO. Based on the equation, the slope value for rGO was 0.0901, while that for CNC–rGO was 0.1331 [47]. Thus, according to the calculation, the *αn* values for rGO and CNC–rGO were 0.66 and 0.44, respectively. For an irreversible electrode process, the *α* values are assumed to be between 0.4 and 0.6 [25,48]. Based on this assumption, the number of electrons transferred (*n*) in the oxidation process can be calculated. Therefore, the numbers of electrons for rGO were equal to 1.64, 1.65, and 1.09 if the *α* values were 0.4, 0.5, and 0.6, respectively. For the nanocomposite CNC–rGO, the *α* values were 1.11, 0.88, and 0.74 for *α* values 0.4, 0.5, and 0.6, respectively. It can be concluded that one electron was involved in the oxidation of MP [49]. The k_0_ value can be determined from the intercept of plot Epa versus log *v* if the E^0′^ is known [50]. The E^0′^ value can be determined by extrapolating to the axis *v* = 0 from the plot of Epa versus *v* as depicted in Figure S8b (Supplementary Materials) [51]. The intercept value from the plot of Epa versus log *v* for rGO was 0.9974, while that for nanocomposite CNC–rGO was 1.0628. Thus, by substituting all the values into Equation (4), the k_0_ value for rGO was determined as 2.918 × 10^3^, while that for CNC–rGO was determined as 1.872 × 10^3^. The k_0_ value demonstrated that the rGO and CNC–rGO modified electrodes promoted the oxidation of MP on the electrode surface. The k_0_ value was comparable to a study by Aliyu et al. that used carbon nanotubes/gold nanoparticles for amoxicillin detection [52]. All the α, k_0_, n, and E^0′^ values are tabulated in Table S2 (Supplementary Materials). 

### 3.4. Optimization of the Nanocomposite CNC-rGO Modified Electrode

All the optimizations and electroanalytical performances of the sensor were carried out using the DPV method. This is because the DPV method gives better reproducibility, sensitivity, and selectivity than the CV method [53,54,55]. The amounts of CNC and rGO for nanocomposite formation could affect the response of the sensor. The current response was the highest when the ratio of CNC to rGO was 2:1, owing to the good film-forming property of CNC that produces a well-distributed nanocomposite on the electrode surface, as shown in Figure S9a (Supplementary Materials) [56]. Based on the observation, the same amount of CNC and rGO (1:1) produced nanocomposites that were not well distributed on the electrode. Peak current was reduced when the ratio was 3:1 (CNC to rGO), probably due to the insulating properties of CNC that reduced the conductivity of rGO [57]. As can be seen in Figure S9b (Supplementary Materials), the peak current dropped to almost half of the original signal when the amount of nanocomposite CNC–rGO was doubled on the electrode surface. The excessive amounts of CNC–rGO nanocomposite on the electrode surface may block the active surfaces, which in turn will slow down the electron transfer process [58].

**Figure 5:**
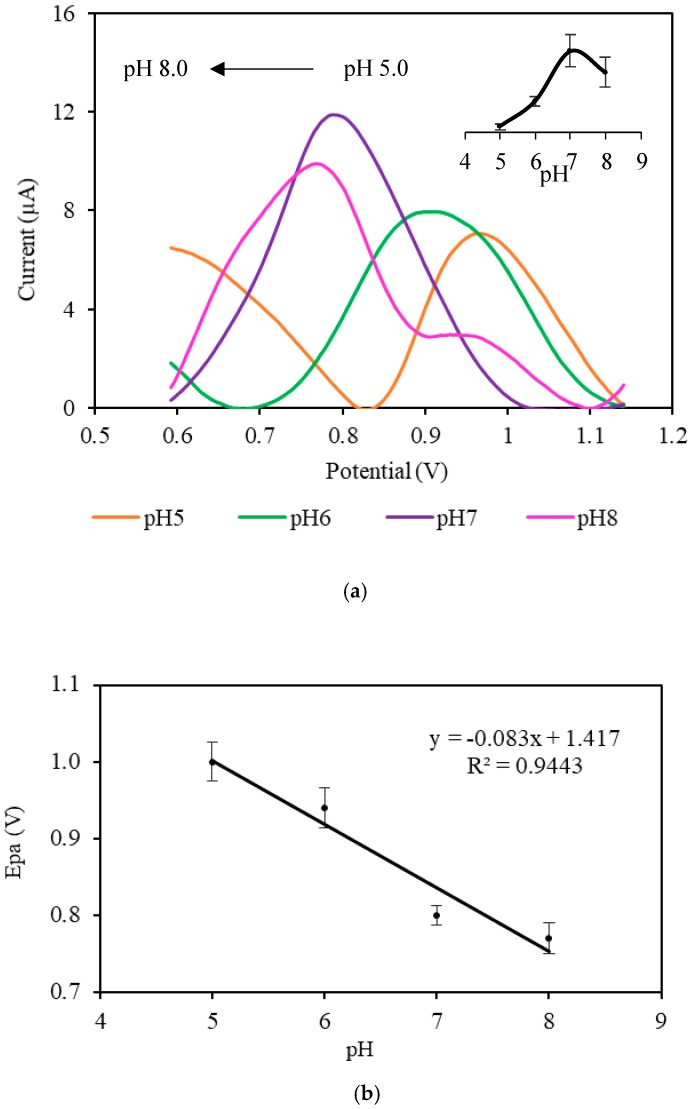
(**a**) Differential phase voltammetry (DPV) voltammograms of CNC–rGO toward 5 × 10^−3^ M MP in PBS (pH 5.0–pH 8.0) at a scan rate of 0.01 V/s. The inset shows the effect of pH on the MP response in the pH range of 5–8; (**b**) plot of oxidation potential versus pH at a scan rate of 0.01 V/s.

Figure 5a displays the DPV response toward MP solution at different pH values (pH 5.0, pH 6.0, pH 7.0, and pH 8.0). The peak potential shifted to the less positive potential when the pH was increased from pH 5.0 to pH 8.0, indicating that the oxidation of MP was involved in the transfer of protons. Figure 5b shows the plot of oxidation potential (Epa) versus pH. It can be clearly seen that the oxidation potential decreased linearly with pH where the corresponding equation was Epa = −0.083*x* + 1.417 (*R*^2^ = 0.9443). The slope value was about −0.083 V∙pH^−1^, which is larger than the value of the Nernst equation (−0.059 V∙pH^−1^) for a two proton–two electron process [59]. Paraben, specifically MP, having a pKa value of 8.2, is present in the protonated and deprotonated forms at different pH values. At pH less than 3.0, paraben is in the protonated form, while, in the pH range of 4.0–6.5, paraben is in a neutral form. In the range of pH 7.0–9.0, paraben is mostly negatively charged because it is in the deprotonated form [60,61]. The inset for Figure 5 displays the current response versus pH, and it shows that pH affected the oxidation of MP. The oxidation peak increased when the pH increased from pH 5.0 to pH 7.0; however, the current decreased at pH 8.0. The same finding was found by Dai et al. in electrochemical detection of triclosan. They found that the current was reduced at pH 8.0 due to the phenolic dissociation, which subsequently produced anions. The highest anodic response was found at pH 7.0. Hence, pH 7.0 was used for subsequent experiments [62].

**Figure 6:**
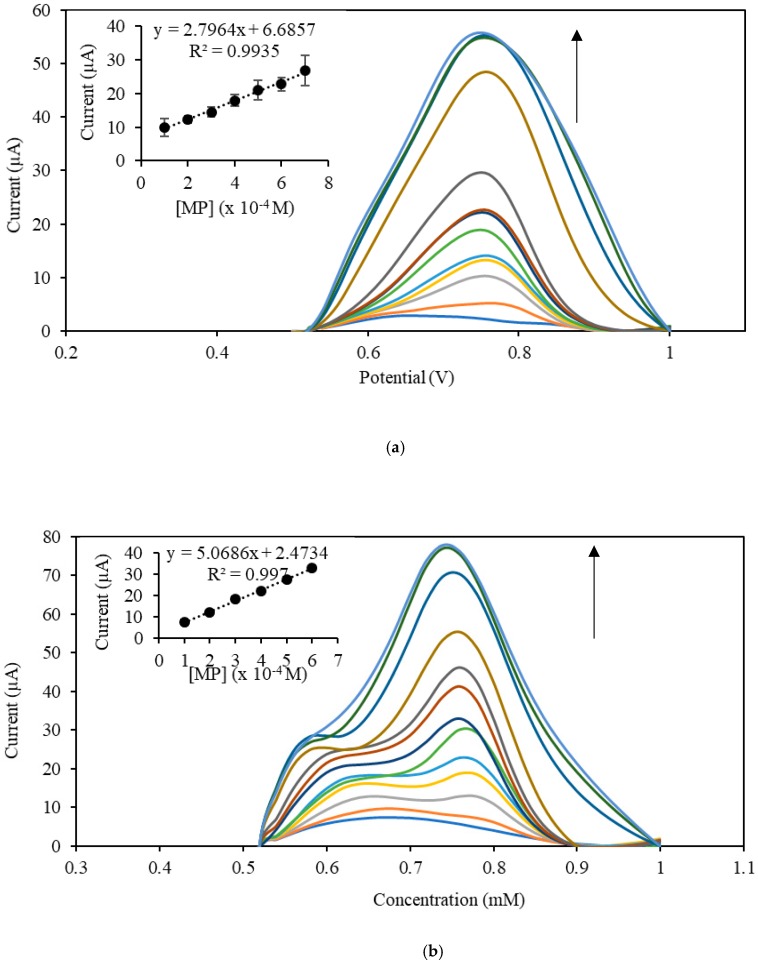
DPV voltammograms of (**a**) CNC–rGO nanocomposite and (**b**) rGO modified electrodes toward different concentrations of MP. The inset shows the linear calibration curve from 2 × 10^−4^–9 × 10^−4^ M MP at 0.75 V (0.05 M PBS pH 7.0).

A series of different concentrations of MP was prepared to study the effect of different concentrations on the DPV peak current at pH 7.0 (0.05 M). Under optimal conditions, both CNC–rGO nanocomposite and rGO modified electrodes displayed that the current increased proportionally to the concentration of MP, as illustrated in Figure 6a. The linear response was in the range of 2 × 10^−4^–7 × 10^−4^ M with an *R*^2^ value of 0.9935 and an LOD of 1 × 10^−4^ M for CNC–rGO nanocomposite. However, the rGO modified electrode (Figure 6b) gave a linear response in the range of 1 × 10^−4^–6 × 10^−4^ M with an LOD of 2.6 × 10^−5^ M. The sensitivities of both rGO and CNC–rGO nanocomposite modified electrodes were calculated from the calibration curve slope and divided by the electroactive working area of the electrodes [63]. The calculated sensitivity values were found to be 26.8 µA·mM^−1^·cm^−2^ and 19.2 µA·mM^−1^·cm^−2^ for rGO and CNC–rGO nanocomposite modified electrodes, respectively.

#### 3.4.1. Reproducibility, Repeatability, and Stability Studies

The reproducibility of both rGO and nanocomposite CNC–rGO modified electrodes was measured using five different sensors in 4 × 10^−4^ M MP, and the results are displayed in Figure S10 (Supplementary Materials). The RSDs for both rGO and CNC–rGO nanocomposite were found to be 21.04% and 8.20%, respectively. The CNC–rGO nanocomposite modified electrode showed acceptable reproducibility, as the RSD value was less than 10% [64]. As for the rGO modified electrode, the RSD value was quite high, which may be due to the competition between the adsorption of MP and direct oxidation of MP on the rGO surface, which may lead to lower reproducibility. 

**Figure 7:**
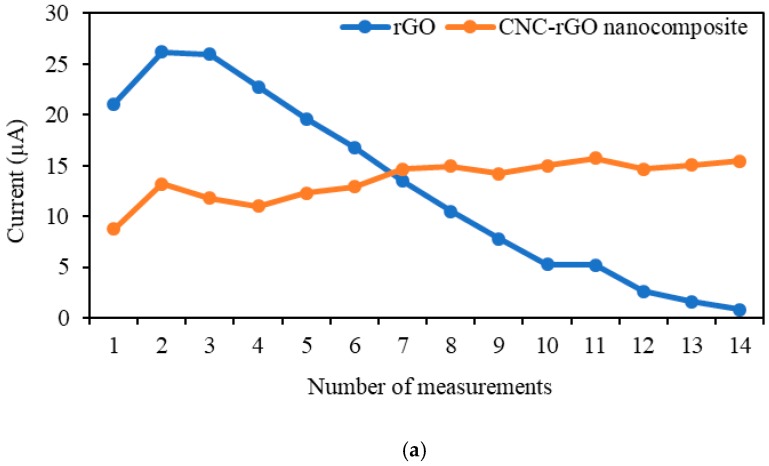

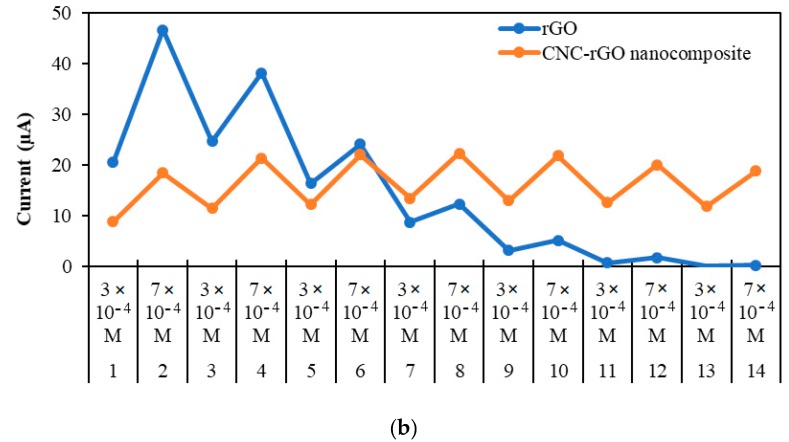
Repeatability study (**a**) rGO and CNC–rGO nanocomposite modified electrodes for 14 consecutive measurements in 3 × 10^−4^ M MP, and (**b**) using one single electrode alternating between 3 × 10^−4^ and 7 × 10^−4^ M MP for rGO and CNC–rGO nanocomposite. All measurements were fixed at 0.75 V.

The repeatability study demonstrated that the same rGO and CNC–rGO nanocomposite modified electrodes yielded RSD values of 71.6% and 14.8% for 14 consecutive measurements. In addition, the electrode modified with CNC–rGO nanocomposite could be used for up to 14 consecutive measurements in two different concentrations alternately with an RSD value of 7.7–12.7%, as shown in Figure 7. Although the rGO modified electrode presented a lower LOD and a higher current response toward MP upon initial measurement, the electrode could only be used for the first measurement, the current response dropped linearly with repeated use, and no signal was found after 14 measurements. The RSD value of the rGO modified electrode after 14 consecutive measurements was between 95% and 101%, which was around 7–8 times higher than that for the CNC–rGO nanocomposite modified electrode. This was probably due to the synergistic effect between CNC and rGO, which improved the characteristic of the nanocomposite form. In a study, Sadasivuni et al. found that the characterization of current–voltage for a CNC/iron oxide composite provided a relatively higher response than the composite that contained iron oxide only [16]. The reduction in the current response of rGO also may be attributed to the ability of rGO to adsorb MP. GO has the capability to adsorb aromatic compounds, especially the unoxidized part known as rGO. The adsorption of the paraben compound may have occurred due to hydrophobic interactions (π–π stacking) between the paraben compound and the benzene ring of GO [65].

The stability of the sensor was investigated by measuring the current response in 5 × 10^−3^ M MP for the duration of 28 days. The sensor was prepared and kept in the dark at room temperature. The sensor retained about 95% of the current response after one week of storage, and the response decreased to about 80% after 28 days of storage. This value indicates that the sensor demonstrated good stability [29].

#### 3.4.2. Selectivity Study and Real Sample Analysis

**Figure 8:**
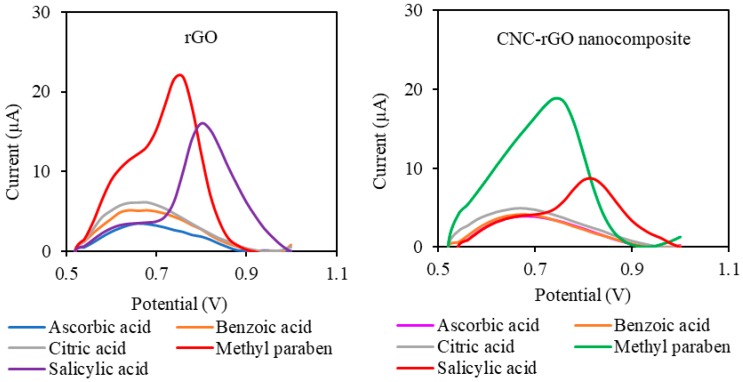
DPV voltammograms of rGO and CNC–rGO nanocomposite modified electrodes toward possible interfering species at a concentration of 4 × 10^−4^ M.

As an electroactive material, paraben determination using the electrochemical method may experience serious interference from other electroactive species. A selectivity study of both rGO and CNC–rGO nanocomposite modified electrodes for MP determination was carried out using a few possible interference substances such as ascorbic acid, benzoic acid, salicylic acid, and citric acid. Ascorbic acid was selected because it is an electroactive species [66], while salicylic acid, benzoic acid, and citric acid may be present in pharmaceutical formulations [67,68,69,70,71]. As can be seen in Figure 8, several common interfering species displayed a much lower current response at potential 0.75 V, while salicylic acid showed a significant peak at potential 0.85 V. Both rGO and CNC-rGO nanocomposite modified electrodes exhibited an almost similar current trend with a slightly higher current in salicylic acid solution. 

Based on *t*-test statistical analysis, the rGO modified electrode demonstrated significant interference due to various interference substances at a 1:10 ratio (Table 1). However, for the CNC–rGO nanocomposite-based electrode, no significant interference from the potential interfering substances was observed, even up to a concentration ratio of 1:10. The absence of interference for the CNC modified electrode may be attributed to the CNC properties. For example, ascorbic acid showed less interference in the presence of CNC, which may be due to the negatively charged CNC surface, which reduces the peak current of ascorbic acid. In a study, He and Zhang demonstrated that the peak current of ascorbic acid was decreased due to the negatively charged surface of multi-walled carbon nanotubes [72].

**Figure 9:**
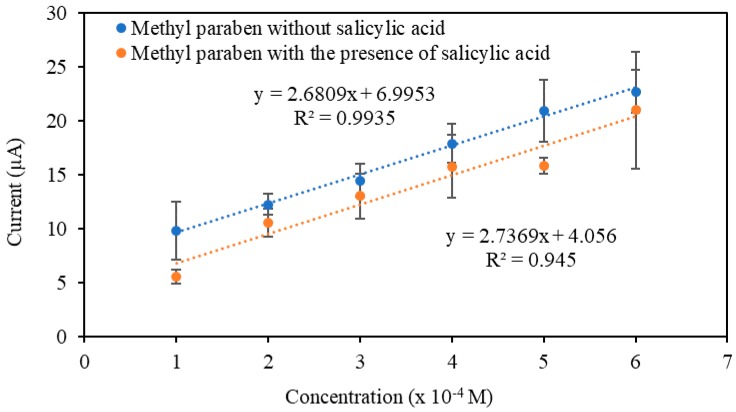
Linear calibration plots of CNC–rGO in MP solution with and without the presence of salicylic acid (SA) solution. SA solution was added only at two concentrations, i.e., 3 × 10^−4^ M and 5 × 10^−4^ M for MP concentrations of 3 × 10^−4^ M and 5 × 10^−4^ M, respectively.

A study comparing the linear calibration plots of MP with and without the presence of salicylic acid at two different concentrations (3 × 10^−4^ and 5 × 10^−4^ M) confirmed that there were no significant changes in the linear response range (Figure 9). Based on the *t*-test statistical analysis at 95% confidence level, there was no significant difference between the linear range without any addition of salicylic acid and with addition of salicylic acid (Table 2). Thus, it can be concluded that there was no interference from salicylic acid. 

All the selectivity studies confirmed that the rGO modified electrode suffered from interference species at high concentrations, while CNC–rGO nanocomposite modified electrodes were relatively free from serious interference. This is probably a result of the ability of CNC to reduce adsorption of various substances onto the rGO surface. Additionally, the synergistic effect between CNC and rGO may improve the properties of the nanocomposite as compared to the single material.

The electrochemical sensor was used for detection of MP in real samples (cream for burns), and the recovery was calculated. Two concentrations of MP were spiked into the real sample solution. The recovery value obtained in the range of 83–106% demonstrated good potential for application of this MP sensor in real sample analysis, as presented in Table S3 (Supplementary Materials). 

### 3.5. Comparison of the Developed Sensor with Other Carbon-Based Paraben Sensors

Table 3 displays and compares the performance of some carbon-based electrochemical sensor methods for determining MP. In most similar sensor methods reported, the determination of MP was carried out at potentials around +0.8–1.0 V; however, in this work, a much lower potential was possible and this could have avoided serious interference from easily oxidized species present in the samples [73,74]. Baytak et al. developed an MP sensor with excellent selectivity, but the preparation of the composite involved sonication for 5–6 h [73]. 

The most sensitive carbon-based electrochemical sensor for paraben compounds with an LOD value of 3.50 × 10^−10^ M also suffered from a long preparation step up to 11 h [75]. Rather et al. [10] developed a sensor with a lower oxidation potential, but the stability was retained at 83% for only one week. In our study, the sensor retained its stability (80%) for at least 28 days. In addition, Mendonça et al. also fabricated an rGO-based electrochemical sensor for MP. In their study, the sensor suffered interference from methyl salicylate, compared to our findings in which salicylic acid did not interfere [74]. A higher oxidation potential was found for MP oxidation in another study conducted using an rGO-based sensor. As mentioned above, a more positive oxidation potential may lead to interference from other easily oxidized species, even though the study showed no interference from species frequently used in cosmetic and personal care products [14].

The electrochemical sensor developed by Wang et al. has an almost similar stability with our study. The sensor showed good stability by retaining 97.3% response after one week of storage at 4 °C [51]. In addition, the MP sensor developed here clearly has the advantage for applications in the analysis of samples with higher concentrations of MP (linear detection range for this sensor is 2 × 10^−4^–9 × 10^−4^ M) without the need for extensive sample dilution. For instance, the concentrations of MP in sweet soy sauce from instant fried noodles [76] and in gel formulation [77] were found to be in the range of 7.8 × 10^−4^–1.38 × 10^−3^ M and up to 1.31 × 10^−3^ M, respectively. Furthermore, although the LOD was higher compared to other sensors, the LOD of this sensor (0.0011%, *w*/*w*) was still below the permissible limit set by the Cosmetic Directive. The amount of paraben used was usually less than 0.3%, as the Food and Drug Administration (FDA) limits MP in food products at 0.1%. On the other hand, the concentration of paraben used in pharmaceutical products usually differs for each product, typically not exceeding 1% [78].

## 4. Conclusions

An electrochemical sensor for MP detection was successfully developed. The sensor is the first of its kind that utilizes cellulose nanomaterial for the detection of MP. The sensor showed acceptable reusability, and it was confirmed that the CNC appeared to avoid or reduce the excessive adsorption of MP onto the rGO surface, which led to good selectivity toward MP with very little interference from ascorbic acid. Another advantage of this sensor is the possibility of the determination of higher concentrations of MP without extensive dilution, considering that the permissible levels in various types of samples are <1% when compared with most other reported sensors for MP.

## Figures and Tables

**Table 1:** Comparison of the response of reduced graphene oxide (rGO) and cellulose nanocrystal (CNC)–rGO modified electrodes toward possible interference species at two different concentration ratios. MP—methyl paraben.

Interference	Current (µA)							
	rGO				CNC–rGO Nanocomposite			
	1:1	t_calculated_	1:10	t_calculated_	1:1	t_calculated_	1:10	t_calculated_
MP	23.43 ± 1.36	-	36.10 ± 1.41	-	18.11 ± 2.99	-	18.81 ± 1.65	-
MP + ascorbic acid	29.22 ± 1.58	1.000	29.59 ± 2.40 *	2.97	17.03 ± 1.20	1.05	11.83 ± 1.44	1.33
MP + salicylic acid	25.32 ± 6.30	0.590	26.86 ± 3.56 *	3.24	17.22 ± 0.55	0.47	20.51 ± 1.21	1.18
MP + benzoic acid	23.41 ± 4.12	0.005	33.10 ± 0.62 *	5.59	17.34 ± 1.74	0.30	15.47 ± 1.35	1.41
MP + citric acid	23.61 ± 2.29	0.086	34.81 ± 3.22	0.74	16.82 ± 0.38	0.36	16.85 ± 1.92	2.35

Note: If t_calculated_ > t_critical_, * indicates a significant difference where T_critical_ = 2.92 (95% confidence level).

**Table 2:** Comparison of point-based linear range of MP without any addition of salicylic acid and with addition of salicylic acid at only two different concentrations, 3 × 10^−4^ M and 5 × 10^−4^ M.

Linear Range without Any Addition of Salicylic Acid		Linear Range with Addition of Salicylic Acid		*t_calculated_
Concentration	Current (µA) ± SD	Concentration	Current (µA) ± SD	
3 × 10^−4^ M MP	12.27 ± 1.00	3 × 10^−4^ M MP + 3 × 10^−4^ M SA	10.56 ± 1.28	1.32
5 × 10^−4^ M MP	17.93 ± 1.78	5 × 10^−4^ M MP + 5 × 10^−4^ M SA	15.80 ± 2.88	0.84

Note: * *t*-test; t_critical_, t = 2.92 (95% confidence level).

**Table 3:** Carbon-based electrochemical sensors for paraben detection. LOD—limit of detection; DPV—differential phase voltammetry; EP—ethyl paraben.

Analyte	Detection Method (V)	Sensing Materials	Linear Range (mM)	LOD (mM)	Reference
MP	DPV (+0.75)	Nanocomposite CNC–rGO	2.00 × 10^−4^ to 9.00 × 10^−4^	1.00 × 10^−4^	This work
MP	Square wave voltammetry (SWV) (+0.768)	Carbon nanofibers (CNFs) and nickel–cobalt–palladium nanoparticles, (Co-Ni-Pd)NPs-CNFs/GC	3.00 × 10^−9^ to 3.00 × 10^−7^	1.20 × 10^−9^	[73]
EP	SWV (+0.7)	Fullerene nanorod modified glassy carbon electrode (C60NRs–NH–Ph–GCE)	1.00 × 10^−8^ to 5.20 × 10^−7^	3.80 × 10^−9^	[10]
MP	DPV (+0.78)	rGO decorated with ruthenium nanoparticles	5.00 × 10^−7^ to 3.00 × 10^−6^	2.40 × 10^−7^	[74]
MP	SWV (+1.0)	rGO/gold nanoparticle (AuNP) nanocomposite	3.00 × 10^−8^ to 1.30 × 10^−6^	1.38 × 10^−8^	[14]
EP	SWV (+0.76)	Composite of CNFs and tri-metallic nanoparticles of gold, cobalt, and nickel (Au-Ni-Co)NPs-CNFs/GCE	1.00 × 10^−9^ to 1.00 × 10^−7^	3.50 × 10^−10^	[75]
MP	Linear sweep voltammetry (LSV) (>+1.0)	Langmuir–Blodgett (LB) film of multi-walled carbon nanotubes (MWCNTs) perpendicularly modified glassy carbon electrode (GCE), (MWCNTs-LB/GCE),	1.00 × 10^−6^ to 8.00 × 10^−5^	4.00 × 10^−7^	[51]

## Supplementary Materials

The following are available online at https://www.mdpi.com/1424-8220/19/12/2726/s1.
