# Supplementary material for: A New Sensor for Methyl Paraben Using an Electrode Made of a Cellulose Nanocrystal–Reduced Graphene Oxide Nanocomposite
Source: Sensors (Basel). 2019 Jun 18;19(12):2726. doi: 10.3390/s19122726 (PMC6630541; doi:10.3390/s19122726)
Supplement: Supplementary file 1 [file sensors-19-02726-s001.pdf]

A New Sensor for Methyl Paraben Using an Electrode Made of a Cellulose Nanocrystal–Reduced Graphene Oxide Nanocomposite

Wan Elina Faradilla Wan Khalid [1,2], Mohamad Nasir Mat Arip [3], Latifah Jasmani[3] and Lee Yook Heng [1,*]

[1] School of Chemical Sciences and Food Technology, Faculty of Science and Technology, Universiti Kebangsaan Malaysia, 43600 Bangi, Selangor, Malaysia; wan_elina@uitm.edu.my (W.E.F.W.K.); leeyookheng@yahoo.co.uk (L.Y.H.)

[2] Faculty of Applied Science, Universiti Teknologi MARA Negeri Sembilan, Kuala Pilah Campus, Pekan Parit Tinggi, 72000 Kuala Pilah, Negeri Sembilan Darul Khusus, Malaysia

[3] Forest Products Division, Forest Research Institute Malaysia, Selangor 52109, Malaysia; mnasir@frim.gov.my (M.N.M.A.); latifah@frim.gov.my (L.J)

* Correspondence: leeyookheng@yahoo.co.uk; Tel.: +603-89213356; Fax: +603-89215410

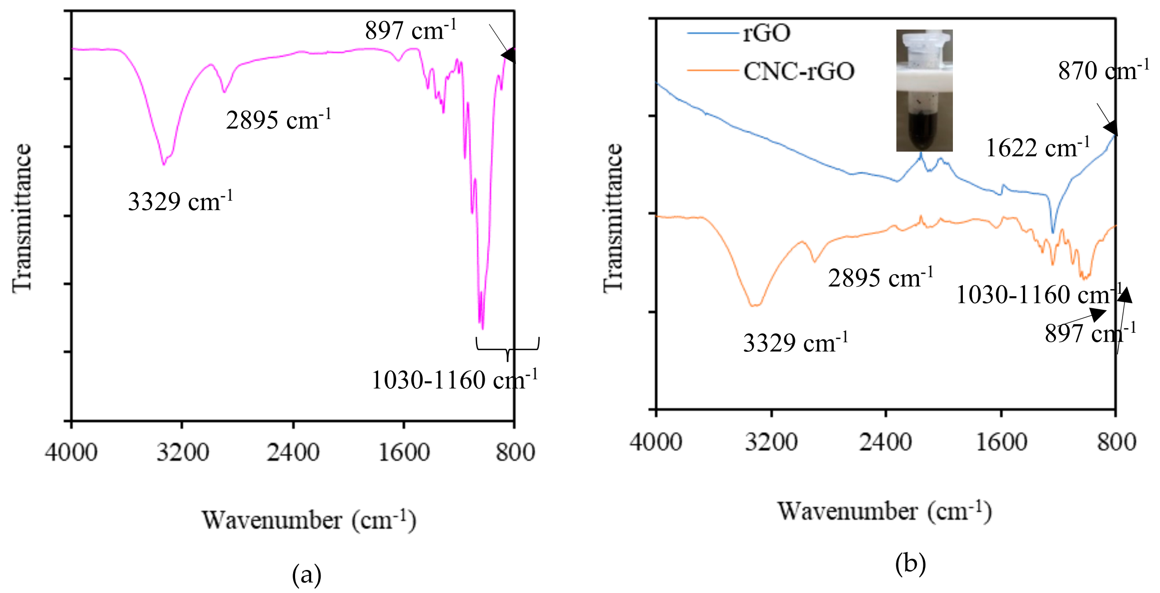

Figure S1. FTIR spectra for (**a**) CNC (**b**) rGO and CNC-rGO. Inset shows the CNC-rGO dispersion after sonication for 1 h.

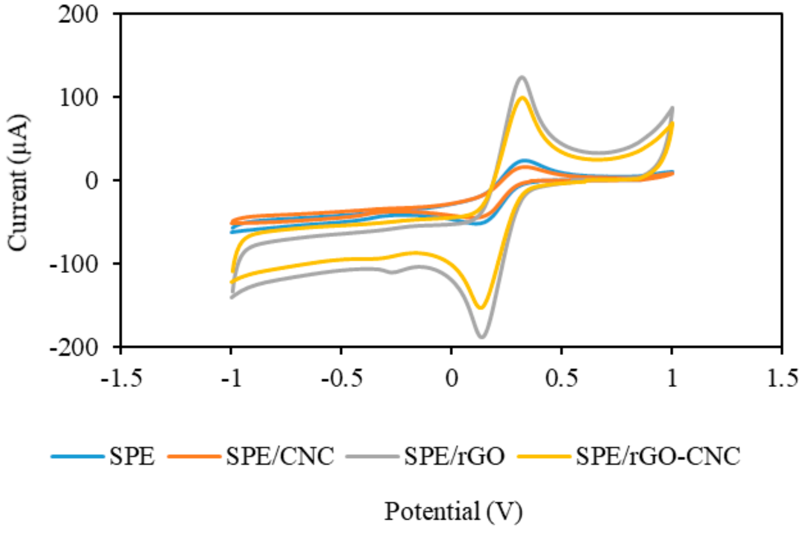

Figure S2. CV of different types of modified electrodes in 10 mM $K_3Fe(CN)_6$ in 0.1 M KCl. Conditions: Scan rate 0.01 V/s, 0.05 M PBS pH 7.0.

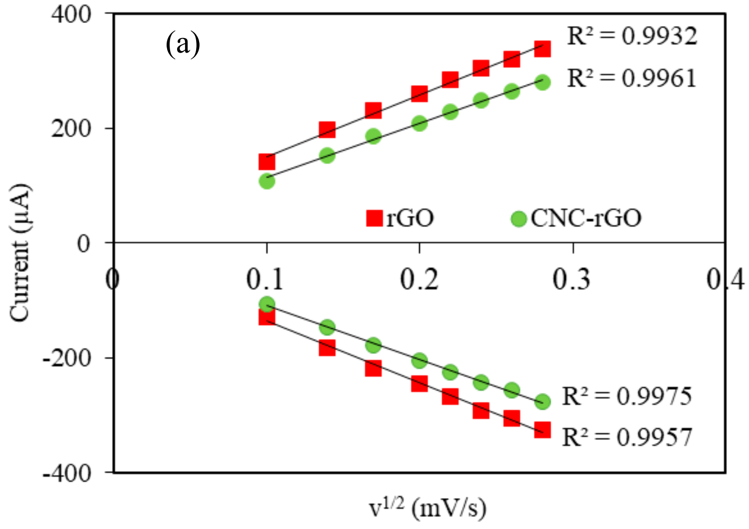

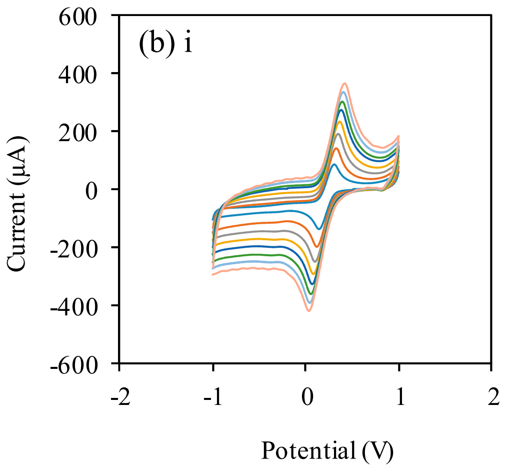

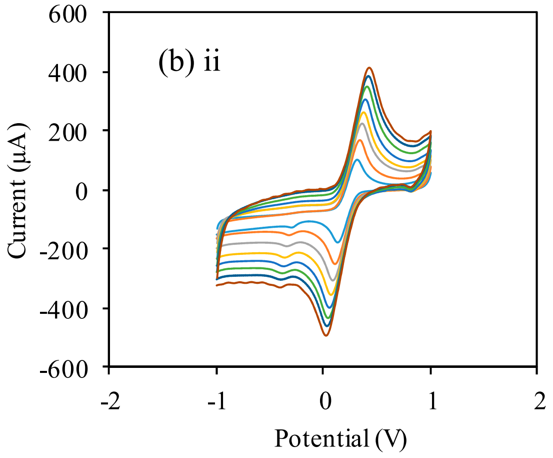

Figure S3. (**a**) Plot of current versus square root of the scan rate for rGO and CNC-rGO (b) CVs from the scan rate (0.01 – 0.08 V/s) for **b**(i) CNC-rGO **b**(ii) rGO in 10 mM $K_3Fe(CN)_6$ in 0.1 M KCl.

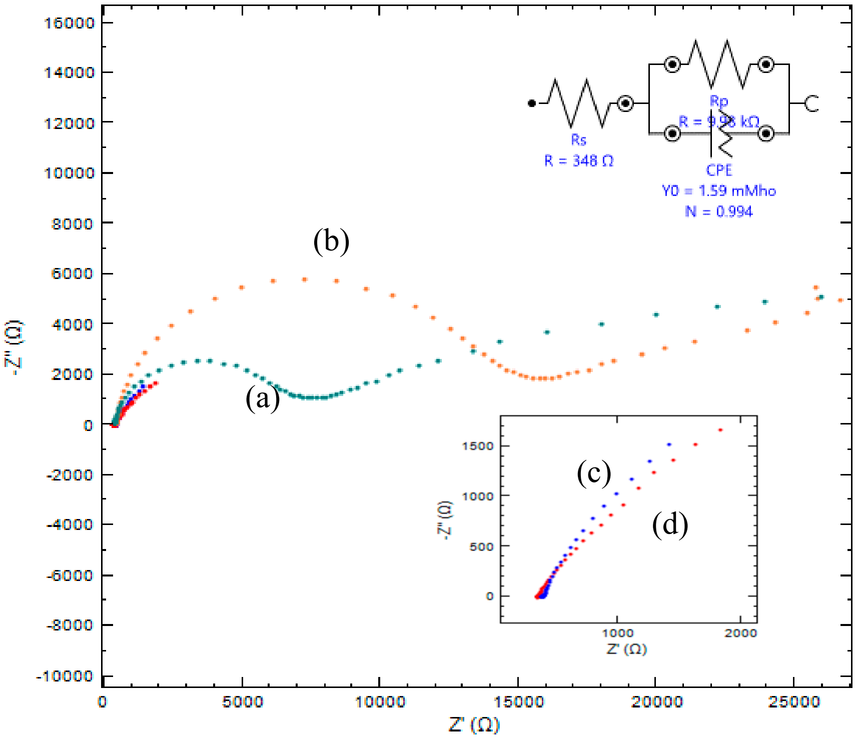

Figure S4. Nyquist plot for (**a**) bare SPE, (**b**) CNC, (**c**) rGO and (**d**) CNC-rGO modified SPE in 10 mM $[Fe(CN)_6]^{3}/[Fe(CN)_6]^{4-}$ solution in 0.1 M KCl. Inset: Equivalent circuit for CNC-rGO modified SPE.

(a)

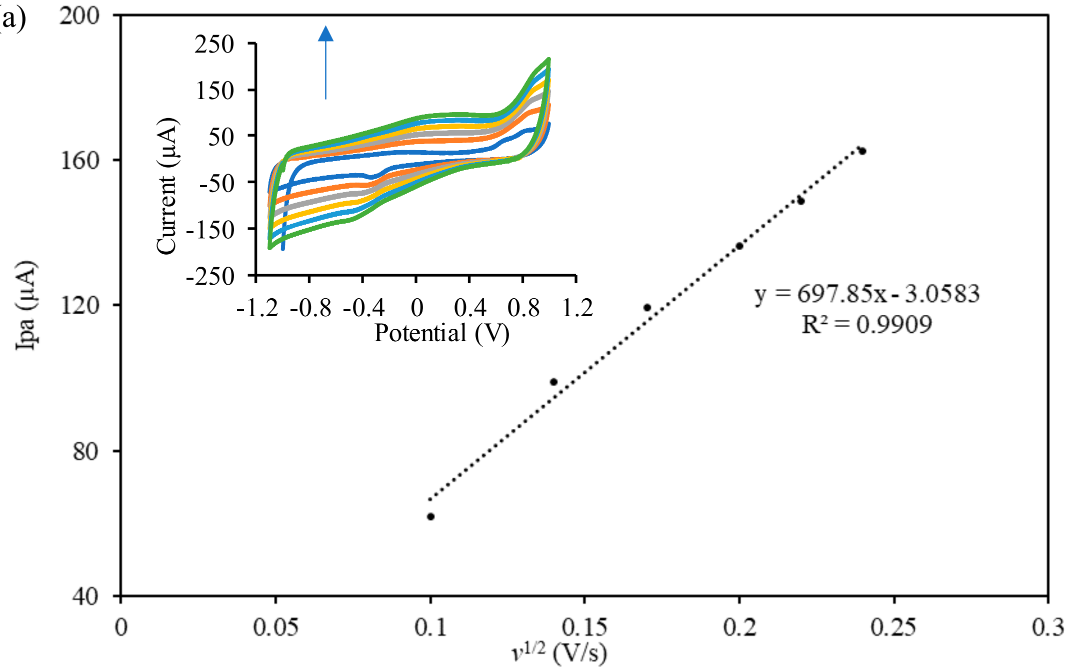

(b)

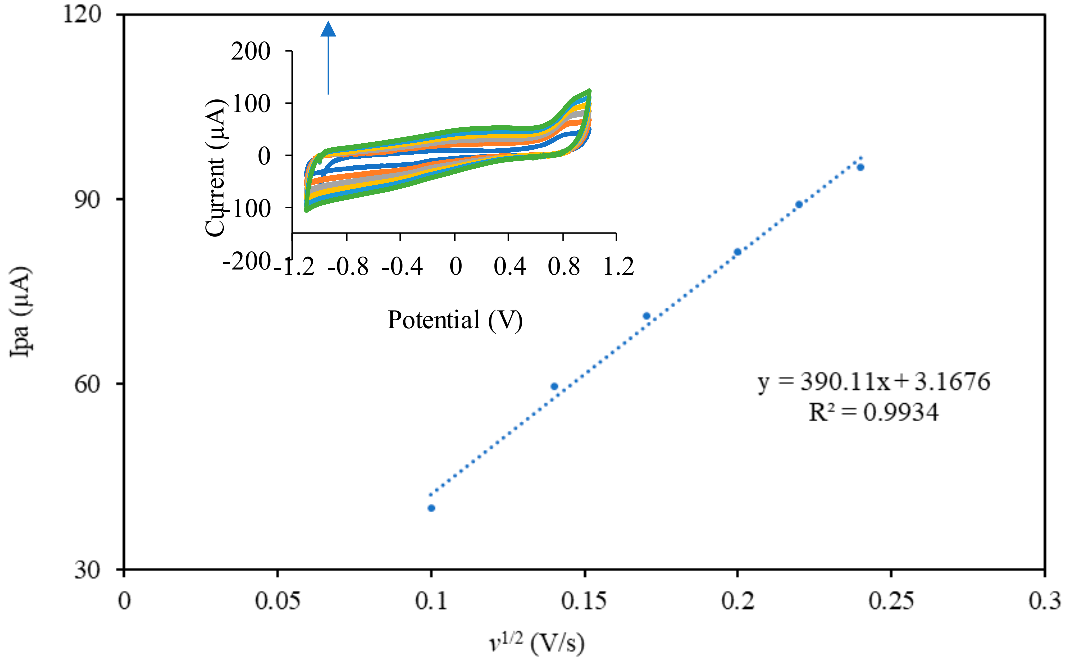

Figure S5. Plot anodic current versus square root of the scan rate for (**a**) rGO modified electrode (**b**) CNC-rGO nanocomposite. Inset shows the CV of the effect of scan rate.

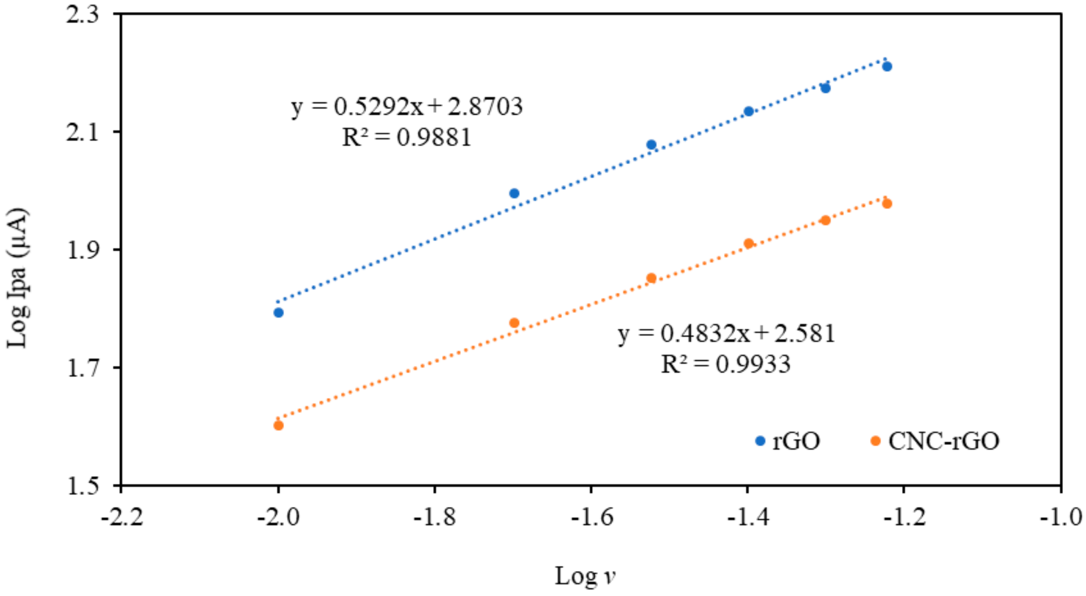

Figure S6. Plot of log Ipa versus log scan rate for rGO and CNC-rGO nanocomposite modified electrodes.

(a)

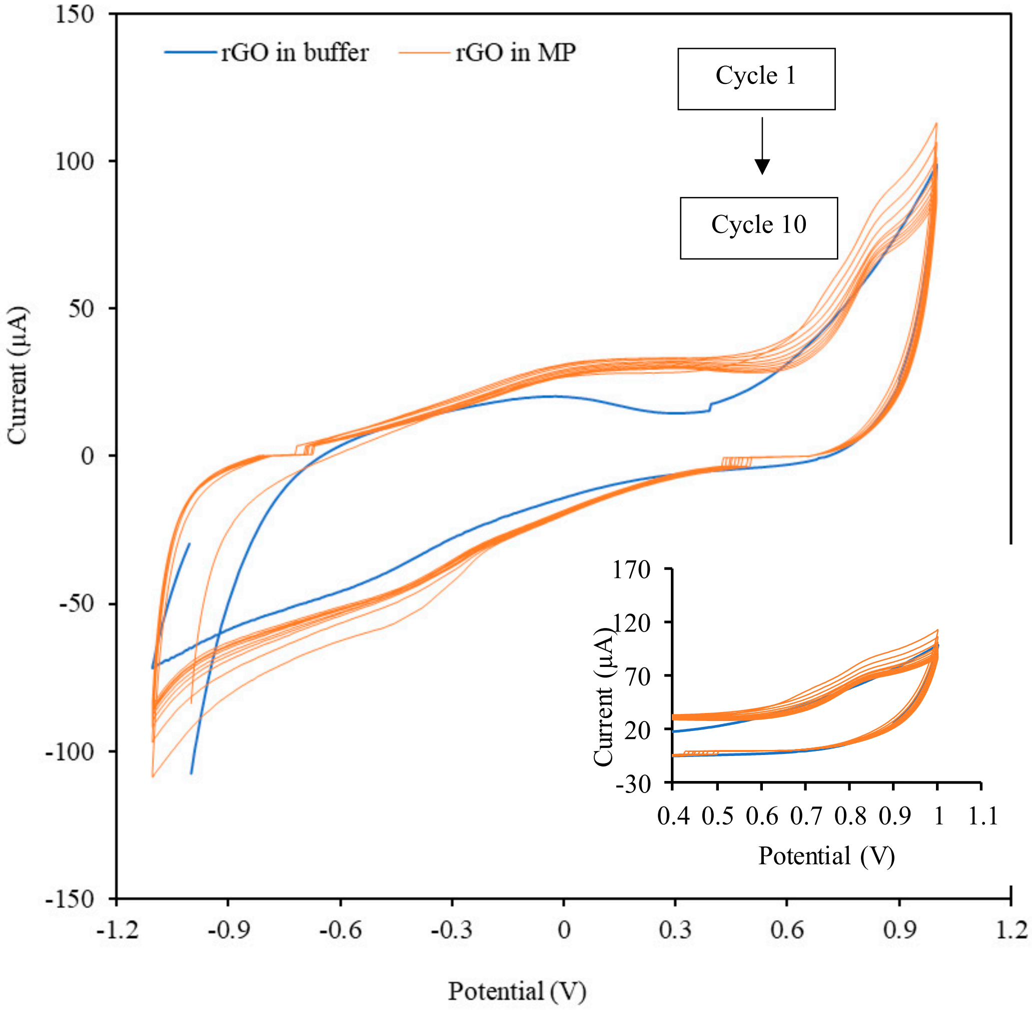
rGO in buffer
rGO in MP
Cycle 1
Cycle 10
Current (μA)
Potential (V)
150
100
50
0
-50
-100
-150
-1.2
-0.9
-0.6
-0.3
0
0.3
0.6
0.9
1.2
170
120
70
20
-30
0.4
0.5
0.6
0.7
0.8
0.9
1
1.1

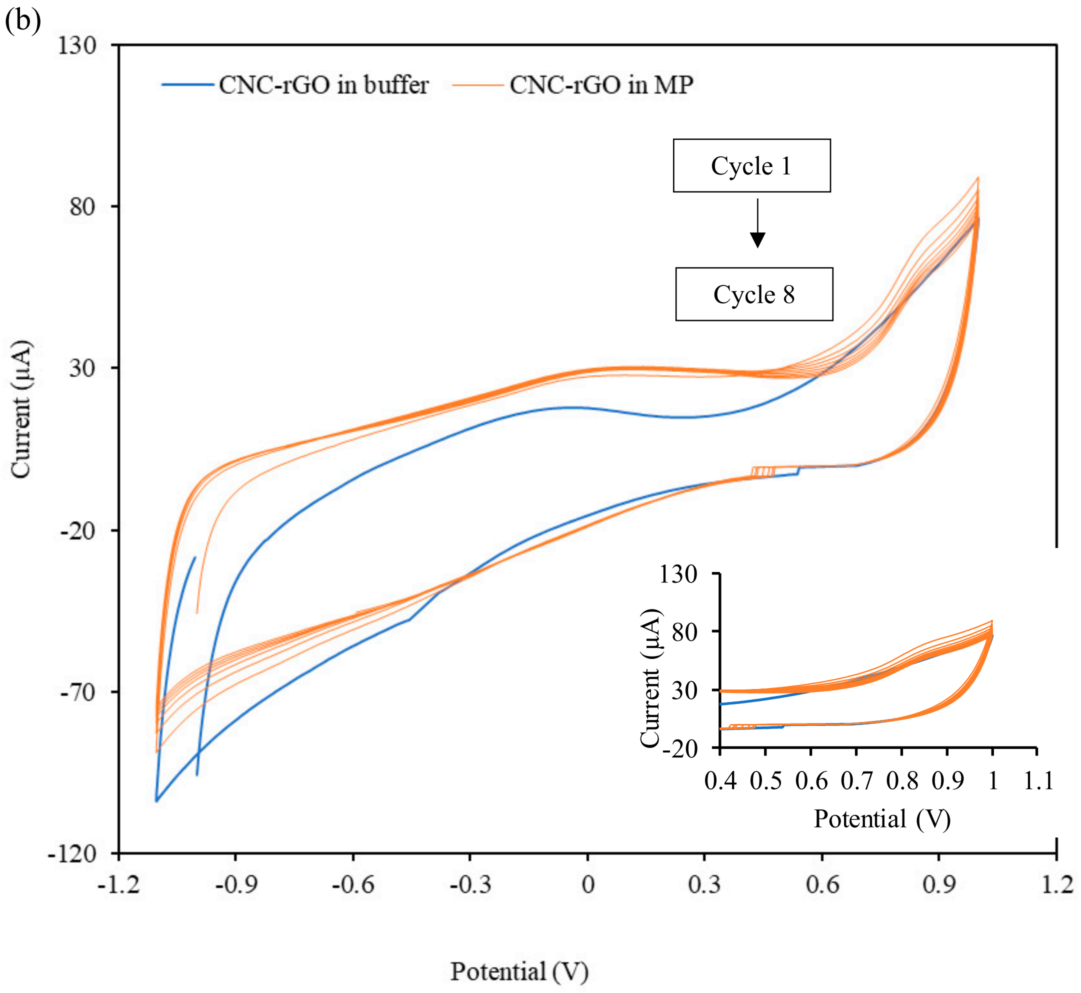

Figure S7. CV of the effect of cycles on (**a**) rGO and (**b**) CNC-rGO nanocomposite response in the presence of 4 × 10^{-4} M MP in K-phosphate buffer pH 7.0 (0.05 M).

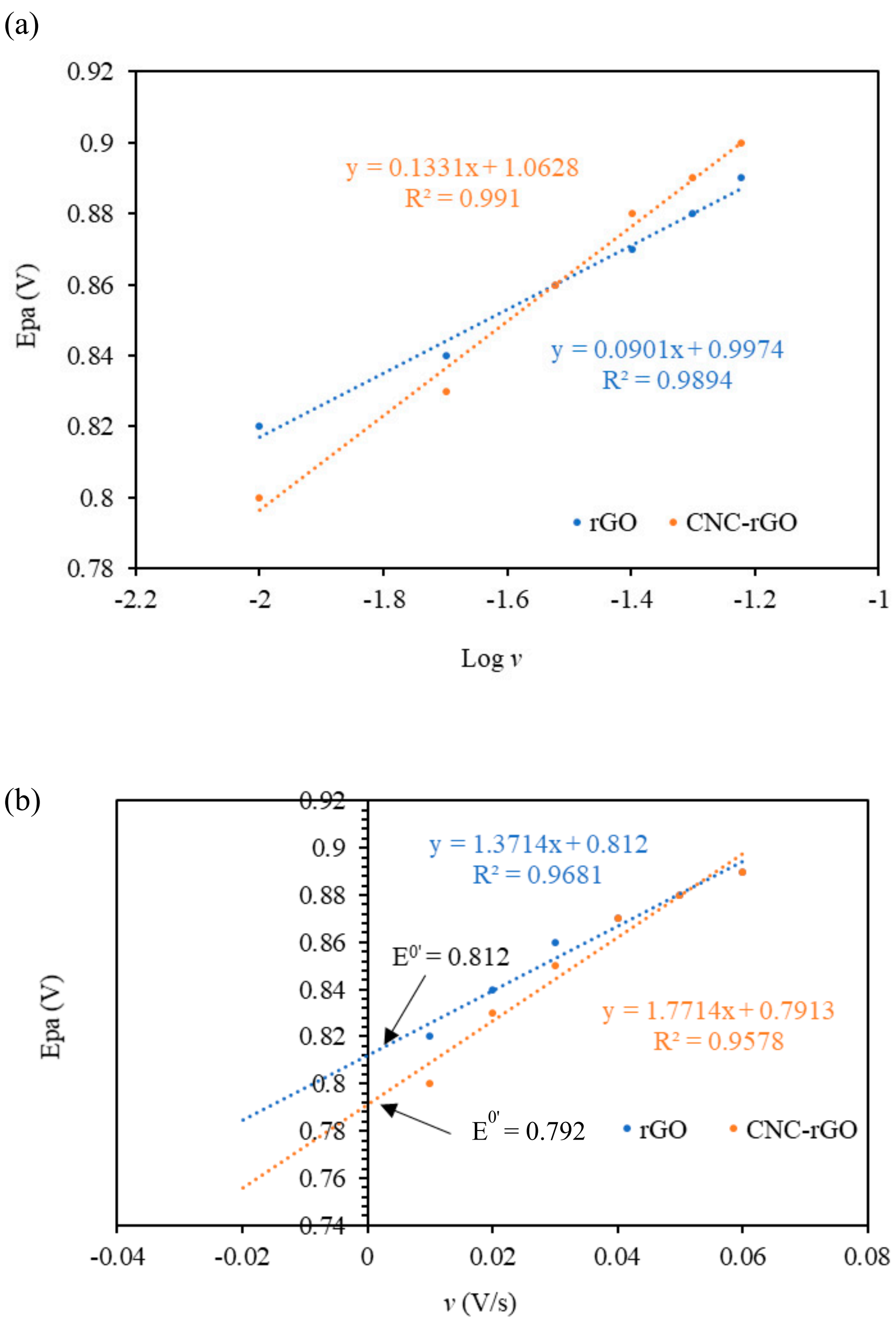

Figure S8. (**a**) Plot of Epa versus log scan rate and (**b**) Plot Epa versus scan rate with $E^{0'}$ value determination by extrapolating the plot to $v = 0$ for rGO and CNC-rGO nanocomposite.

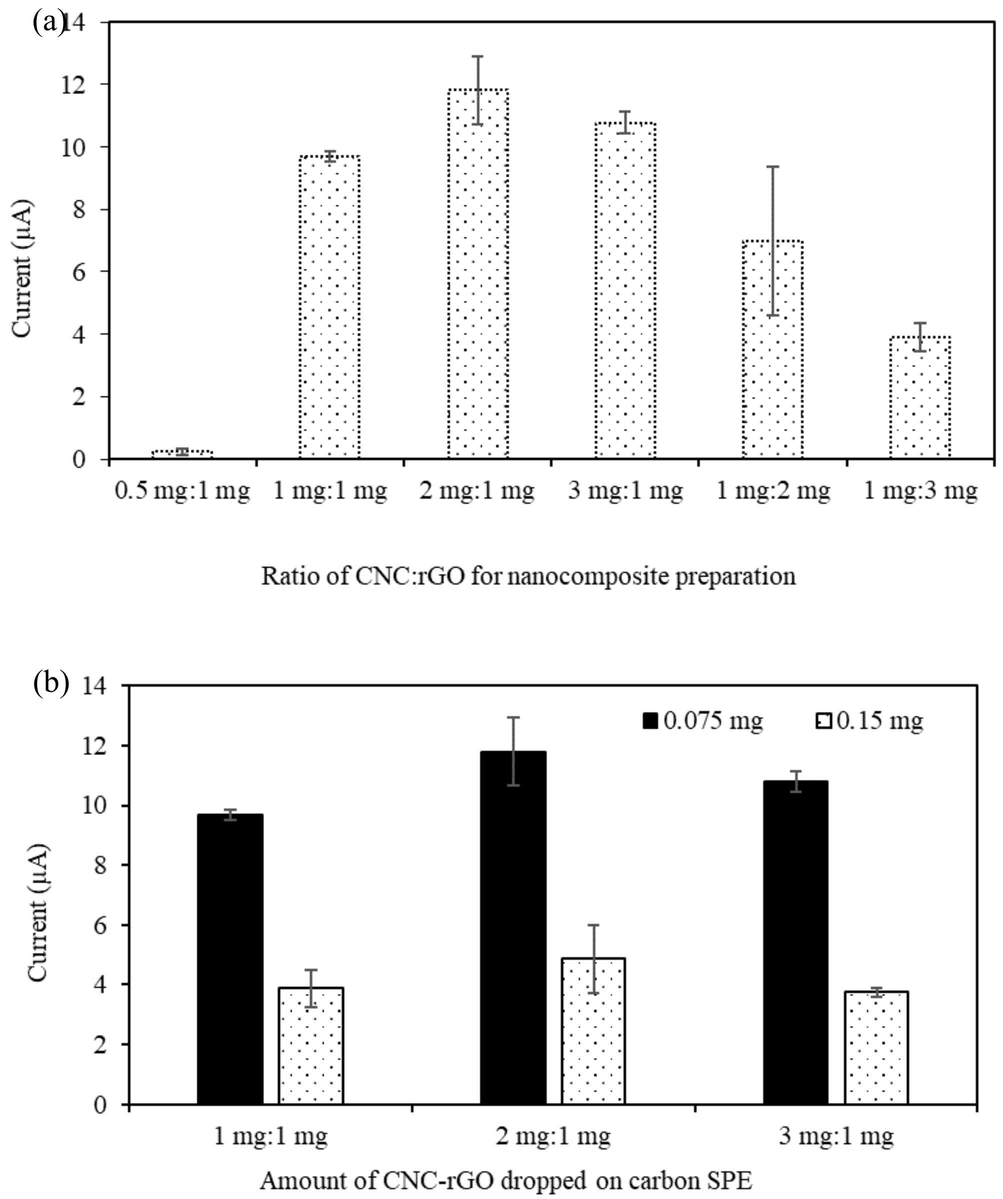

Figure S9. (**a**) Current response of different CNC:rGO weight ratio for CNC-rGO nanocomposite preparation (**b**) Response on the amount of CNC-rGO nanocomposite immobilized on the electrode surface using three different CNC to rGO ratios towards 5 x 10^{-3} M MP *0.075 mg and 0.15 mg refer to amount of CNC-rGO that was dropped on the carbon SPE. Conditions: Scan rate 0.01 V/s; 0.05 M PBS pH 7.0.

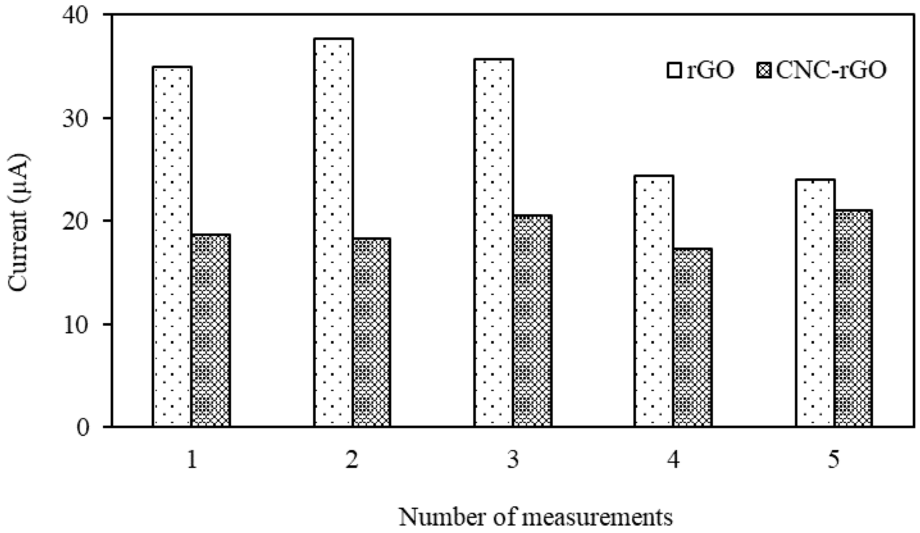

Figure S10. Reproducibility study of rGO and nanocomposite CNC-rGO modified electrodes in 4 x 10^{-4} M MP at 0.75 V.

Table S1. Electrochemical characterization of the modified electrodes in 10 mM $K_3Fe(CN)_6$ in 0.1 M KCl.

Electrodes	ΔEp (mV)	Ipa (µA)
Bare SPE	203	24.41
SPE/CNC	217	16.09
SPE/rGO	175	121.77
SPE/rGO-CNC	198	97.63

Table S2. The value for all the parameter in Laviron equation for rGO and CNC-rGO nanocomposite modified electrode.

Parameters	rGO	CNC-rGO nanocomposite
αn	0.6566	0.4445
k_0	2.918×10^3 s^{-1}	1.872×10^3 s^{-1}
n	1.65	0.889
$E^{0'}$	0.812 V	0.792 V

Table S3. Recovery study of MP spiked in Aloe Vera cream for burn sample.

Sample	Added (M)	Detected[a] (M)	Recovery (%)
1	3×10^{-3}	2.5×10^{-3}	83.3 $\pm$ 0.21
2	5×10^{-3}	5.3×10^{-3}	106.6 $\pm$ 1.28

[a] Number of measurement, n = 3
